# Frequency of Dysnatremia in the First 24 Hours and Its Relationship with Mortality in Patients with Severe Brain Trauma: Dysnatremia and Head Trauma

**DOI:** 10.31661/gmj.vi.3591

**Published:** 2025-08-01

**Authors:** Behrang Rezvani Kakhki, Sara Ghafari Toran, Amir Masoud Hashemian, Reza Akhavan, Maryam Mohammadi, Arman Hakemi, Rohie Farzaneh, Mahdi Foroughian

**Affiliations:** ^1^ Department of Emergency Medicine, Faculty of Medicine, Mashhad University of Medical Sciences, Mashhad, Iran; ^2^ Student Research Committee, Mashhad University of Medical Sciences, Mashhad, Iran; ^3^ Mehrgan Hospital, Kerman, Iran; ^4^ Bahar Hospital, Shahroud University of Medical Sciences, Shahroud, Iran

**Keywords:** Dysnatremia, Mortality, Brain Trauma

## Abstract

**Background:**

Background: Dysnatremia (hypernatremia and hyponatremia) is known to make hospitalized traumatic brain injury (TBI) patients vulnerable to morbidity and mortality. This investigation aimed at observing frequency of dysnatremia in the first 24 hours and its relationship with mortality in patients with traumatic brain injuries.

**Materials and Methods:**

Materials and Methods: This prospective descriptive-analytical study was conducted at Hashminejad Hospital in Mashhad from April 2023 to March 2024. The study sample included patients with severe traumatic brain injuries who referred to the emergency room of Hashminejad Hospital in Mashhad. Information about each patient, including age, sex, Glasgow coma score, blood pressure, plasma sodium, creatinine, serum sugar, calcium, potassium, blood urea nitrogen, and blood pressure at the beginning of admission. Mortality was checked up to 48 hours.

**Results:**

Results: A total of 81 patients with traumatic brain injuries, with an average age of 40.33 ± 19.47 years were included in the study; 85% were male and 15% were female. 32 patients (40%) died and the rest were discharged. Out of a total of 81 patients included in the study, 36 (44.5%) suffered from dysnatremia. 16 patients (19.8%) had hyponatremia and 20 (24.7%) had hypernatremia. The statistical results did not show any significant relationship between the sodium status of patients and their outcome. In the next step, statistical analysis showed that the patient’s sodium level during hospitalization is not a predictor of mortality. Diastolic blood pressure and blood sugar in deceased patients were substantially higher than alive patients. The results showed that the only factor that could substantially predict the death of patients was blood sugar, so that every incremental rise in glucose level increased the chance of death by 1.018 times.

**Conclusion:**

Conclusion: The present study showed that although dysnatremia has no significant relationship with the outcome of brain trauma patients, it needs attention due to its high incidence rate (44.5%) in these patients. In addition, blood sugar was introduced as a factor that could predict the death of patients.

## Introduction

Traumatic brain injury (TBI) is the predominant cause of mortality and morbidity worldwide between the all types of traumatic injuries. In the United States, every year, more than 50 thousand of fatalities is resulted from TBI and is estimated to cost $48.3 billion annually [1]. Various investigations have observed the incidence of substantial electrolyte disequilibrium (abnormal increases or decreases in electrolyte levels) in these patients [2]. For instance, elevated sodium levels and decreased calcium levels can manifest in about half of TBI patients, respectively. The pathophysiological mechanism precipitating ion disequilibrium is intricate and multifaceted. A synergy of catecholamine release, hypertonic fluid administration, restricted hydration, and trauma-induced central diabetes insipidus collectively all contribute to electrolyte imbalances [3]. Cholakkal et al. reviewed the literature on sodium imbalances in TBI and found that hyponatremia had an adverse effect on short-term outcomes [4]. Patients with severe TBI are at risk of developing dysnatremia during their intensive care unit (admissions are often necessitated by underlying conditions that predispose patients to complications, including sensory disruptions, central diabetes insipidus accompanied by excessive urine production, and elevated evaporative fluid loss. Additionally, mannitol and hypertonic saline are often used in these patients to reduce cerebral edema and intracranial pressure [5]. Brain death might be inevitable in some circumstances. Hemodynamic, electrolyte, and hormonal disturbances may occur in these patients. So, recognition of these disturbances is crucial for prevention and treatment to ensure patient survival. It is well-established that derangements in cardiovascular dynamics, coagulation pathways, electrolyte balances (notably sodium), glycemic control, and hormonal homeostasis can precipitate substantial mortality risk in individuals with cerebral trauma., identifying these disturbances is essential for prevention and treatment [5]. As an electrolyte imbalance, elevated sodium levels, affecting approximately 6-9% of patients, are correlated with a heightened risk of mortality [6]. Elevated sodium levels can arise from two primary causes: systemic fluid depletion or central diabetes insipidus [7]. If hypotonic fluids are administered to a patient with brain injury, especially during the postoperative period when AVP levels increase as part of the stress response, iatrogenic hyponatremia can occur. In patients with brain injury, hyponatremia may occur due to the syndrome of inappropriate antidiuretic hormone secretion (SIADH) or cerebral salt wasting syndrome (CSWS). Diagnosing these two conditions can be challenging. Both are characterized by hypotonic hyponatremia and high urine sodium concentration. The best way to diagnose them is to evaluate the patient's volume status carefully [8][9]. A study by Ngatuvai et al. in 2023 investigated the association between electrolyte levels and outcomes in patients with brain injury. The study found that hypernatremia had a more robust correlation with fatality in individuals with traumatic brain injury compared to hyponatremia. Forty studies were evaluated in this review. Most studies that examined the association of sodium imbalances and TBI did not find a disparity in death rates between the low-sodium and high-sodium cohorts. In contrast, most studies that evaluated hypernatremia found a significant increase in mortality in hypernatremic subjects. Nevertheless, numerous investigations incorporated individuals with iatrogenic hypernatremia resulting from hypertonic intervention [1]. Kolmodin et al. also discovered that hypernatremia was linked to elevated fatality risk in individuals with traumatic brain injury [2]. Given the review of previous studies that examined the association between dysnatremia and mortality in patients with TBI, it was found that the results of the studies have not yet reached a general consensus, and there are still many contradictions. Despite the importance of the topic, a study on this topic has not been conducted in the emergency department of a hospital. Therefore, the current investigation sought to examine the frequency of dysnatremia in the first 24 hours and its association with mortality.

## Materials and Methods

**Table:** Table1. Demographics and Clinical Findings of Severe TBI Patients at Arrival

**Characteristic**		**Frequency/mean**	**Percentage/SD**
**Age**		40.33	19.47
**Sex**	**Male**	69	85
	**Female**	12	15
	**3**	16	19.8
	**4**	8	9.9
**GCS**	**5**	12	14.8
	**6**	13	16
	**7**	11	13.6
	**8**	21	25.9
**Systolic Blood Pressure **		130.9	30.75
**Diastolic Blood Pressure **		78.1	19.43
**Sodium**		140.99	6.76
* **Normal**		45	55.5
* **Hyponatremia**		16	19.8
* **Hypernatremia**		20	24.7
**Potassium**		4.01	0.63
**BUN**		34.33	17.94
**Creatinine**		1.17	0.41
**Blood Sugar **		165.28	81.04
**Calcium**		8.75	0.78
**CPK**		417.1	294.45
**Outcome**	**Alive**	49	60
	**Dead**	32	40

This prospective descriptive-analytical study was conducted from March 2023 to February 2024 at Hasheminejad Hospital in Mashhad, Iran. The study was approved by the Ethics Committee of our university on November 2, 2022, and code IR.MUMS.REC.1401.389.

### Participants

The research cohort comprised individuals with severe traumatic brain injury who were triaged to the acute care unit of Hasheminejad Hospital in Mashhad.

### Outcome Measures

The primary outcome measures in the study included several key indicators of patient health and electrolyte balance, specifically: Glasgow Coma Scale (GCS) score to assess level of consciousness, blood pressure, serum sodium level, creatinine level, blood glucose level, calcium level, potassium level, and blood urea nitrogen (BUN) level, as well as serum sodium level, which was a repeated measure to monitor changes in sodium levels over time.

After obtaining approval from the Ethics Committee of MUMS, the study was conducted on patients with severe traumatic brain injury who were referred to Hasheminejad Hospital from March 2023 to February 2024. Demographic and clinical data, including age, sex, GCS score, blood pressure, serum sodium level, creatinine level, blood glucose level, calcium level, potassium level, and BUN level, were collected and recorded in a data form at admission and daily thereafter.

Traumatic brain injury was stratified based on the Glasgow Coma Scale (GCS) score into three tiers: moderate [9][10][11][12], minor [13][14][15], and critical [3][4][5][6][7][8]. Elevated sodium levels were defined as a serum sodium concentration exceeding 145 mEq/L, which was further subdivided into three categories: borderline (145-154), moderate (155-165), and extreme (>165). Low sodium levels were defined as a serum sodium concentration below 135 mEq/L, which was further subdivided into three categories: mild (135-125), moderate (125-115), and severe (<115). Survival outcomes were evaluated up to 48 hours post-injury.

### Statistical Analysis

The findings of this investigation were scrutinized using summary statistics (median and interquartile range) and inferential tests, including contingency tables and logistic regression, utilizing IBM SPSS Statistics version 16 (IBM Corp., Armonk, NY, USA). A probability threshold of 0.05 was applied to all calculations.

## Results

**Table:** Table2. Comparison of Cohort of Deceased and Alive Patients for Study Variables

**Characteristic**	**Alive, Frequency (%) or Mean ± SD**	**Dead, Frequency (%) or Mean ± SD**	**P value**	**OR**	**95% CI**
n	48	22	-		
Sodium Status			0.752		
* Hyponatremia	7 (53.8)	6 (46.2)		0.74	(0.24, 2.29)
* Normal Sodium	31 (63.3)	18 (36.7)		1.42	(0.73, 2.78)
* Hypernatremia	10 (55.6)	8 (44.4)		0.79	(0.31, 2.04)
Systolic Blood Pressure	126.78 ± 27.78	139.34 ± 33.28	0.058	1.003	0.975-1.102
Diastolic Blood Pressure	74.70 ± 17.3	83.66 ± 21.3	0.044	1.018	0.975-2.366
Sodium	140.50 ± 6.1	141.97 ± 7.62	0.343	1.012	0.930-1.102
Potassium	3.91 ± 0.43	4.18 ± 0.83	0.057	0.823	0.286-2.366
BUN	31.33 ± 19.27	39.22 ± 14.97	0.054	0.988	0.950-1.028
Creatinine	1.05 ± 0.29	1.36 ± 0.49	0.002	8.489	0.903-79.769
Blood Sugar	134.21 ± 38.95	213.48 ± 104.74	<0.001	1.018	1.007-1.029
Calcium	8.87 ± 0.74	8.56 ± 0.81	0.091	0.575	0.264-1.253
CPK	378.06 ± 259.55	482.44 ± 324.5	0.122	NE	NE

Not estimated, NE.

A total of 81 individuals with critical head trauma, with a mean age of 40.33±19.47 years, were enrolled in the study. Of these, 69 patients (85%) were male and the remaining were female. The lowest GCS score of patients at admission was 3 (19.8%), and the highest was 8 (25.9%). The average arterial systolic and diastolic pressure of individuals upon arrival were 130.90 and 78.10 mmHg, respectively (Table-1). The laboratory findings of patients at admission, comprising electrolytes, potassium, urea nitrogen, serum creatinine, glucose, calcium, and muscle enzyme levels are also presented in Table-1. The serum sodium levels of patients were categorized, and 45 patients (55.5%) had normal sodium levels, while 36 patients (44.5%) had dysnatremia (Table-1). Unfortunately, 32 patients (40%) ultimately died (Table-1). The study compared the characteristics of patients who were alive (n=48) versus those who were dead (n=22, Table-2). The logistic multivariable regression was conducted by adjustment for all study variables. After adjusting for potential confounding variables with a cutoff point of 0.1, it was shown that the patient's sodium level at admission did not predict mortality. There were no significant differences in sodium status, with similar frequencies of hyponatremia, normal sodium, and hypernatremia between the two groups. However, there were significant differences in certain laboratory values, including diastolic blood pressure (P=0.044), creatinine (P=0.002), and blood sugar (P<0.001), with higher values observed in the deceased group. Additionally, systolic blood pressure, potassium, and BUN levels showed trends towards significance, but did not reach statistical significance. Calcium and CPK levels did not differ substantially between the two groups.

## Discussion

The present study aimed to investigate laboratory disorders, including dysnatremia, in the first 24 hours and clinical disorders in patients with severe traumatic brain injury and their relationship with mortality. After adjusting for potential confounding variables with a cutoff point of 0.1, it was shown that the patient's sodium level at admission did not predict mortality. Logistic regression analysis was performed to identify predictors of patient outcomes. The only factor that could significantly predict patient mortality was blood sugar, such that each unit increase in blood sugar increased mortality by 1.018 times. The study's findings on hypernatremia were inconsistent with previous studies, including Amini et al. (10), which found an association between hypernatremia and mortality, and Sean et al. [11], which found no association between hypertonic saline and hyponatremia and mortality. In contrast, M. Li et al. [12] found that the severity of hypernatremia was associated with mortality. The study's small sample size may have contributed to the inconsistent findings, highlighting the importance of considering multiple factors when evaluating the relationship between laboratory disorders and mortality in patients with TBI. Another study by Rayatdoost et al. in 2022 (13) sought to examine the correlation between electrolyte imbalance at presentation and prognosis in individuals with critical head trauma. This retrospective investigation was conducted on all adult individuals admitted with critical head trauma (GCS 8 or less) at Shahid Beheshti Hospital in Kerman, southwestern Iran. Electrolyte imbalance was the primary endpoint of in-hospital fatality. Persistent vegetative state, severe impairment, moderate impairment, and full recovery were secondary endpoints. Demographic characteristics, vital signs, and mechanism of injury were also documented. Univariate analysis was performed to identify factors associated with fatality. 99 individuals met the inclusion criteria. Ten individuals (10.10%) experienced electrolyte imbalance. There was a significant correlation between electrolyte imbalance and treatment outcome. The risk of fatality in individuals with electrolyte imbalance was significantly higher than in participants with normal electrolyte levels. Full recovery had a lower probability in individuals with electrolyte imbalance compared to participants with normal electrolyte levels. Other outcomes did not differ statistically between the study cohorts [14]. Both investigations were prospective, conducted in the same country with a comparable participant pool, but the findings were not congruent. Electrolyte imbalance was observed in 10% of participants in the earlier investigation, but in 45% of participants in the current study. Although the prevalence of electrolyte imbalance was higher in our study, in contrast to the results of Rayatdoost Doost and colleagues, electrolyte imbalance was not significantly correlated with fatality.

In the present study, dysnatremia was observed in 44.5% of patients, most of whom had mild dysnatremia. However, the incidence of dysnatremia varied in other studies. In Vedantam and colleagues' study, hypernatremia was diagnosed in 36.9% of patients [15]. The incidence of hypernatremia in the present study was significantly lower, at 24.7%. Also, in N. Moro and colleagues' study, hyponatremia had an incidence of approximately 33.0% [16]. This difference may be due to differences in management during admission and treatment. The incidence of hypernatremia in Li and colleagues' study was 28% (12). The incidence of hyponatremia was reported to be 19% in the present study.

The recommendations for the care of severe head trauma include hypertonic therapy, but do not provide specifics on the management and prevention of electrolyte disturbances [16]. In 2020, guidelines for the management of elevated intracranial pressure in individuals with severe head trauma, which recommend that supportive care should include correction of electrolyte abnormalities [17]. However, the mentioned guideline has not provided explicit management protocols for electrolyte disturbances. The Committee on Trauma of the American College of Surgeons has published guidelines for the care of head trauma, which state that the goal of treatment includes maintaining electrolytes within the normal range, with particular attention to sodium levels [18]. Although in the current investigation, electrolyte imbalance was not associated with patient outcomes, these types of electrolyte disorders have a high prevalence, such that in the current study, 44.5% of participants developed electrolyte imbalance.

## Conclusion

The present study showed that although dysnatremia has no significant relationship with the outcome of TBI patients, it needs attention due to its high incidence rate (44.5%) in these patients. Also, the comparison of blood pressure and laboratory findings between deceased and discharged patients showed that diastolic blood pressure and blood sugar were substantially higher in deceased patients than in living patients. The logistic regression test showed that the only factor that could significantly predict the death of patients was blood sugar, so that every incremental rise in glucose level in blood sugar increased the chance of death by 1.018 times.

## Conflict of Interest

The authors declare no conflicts of interest.
